# Anesthesia Management of a Liver Transplant Recipient with Remimazolam

**DOI:** 10.1155/2023/5935657

**Published:** 2023-01-12

**Authors:** Takashi Kawasaki, Takafumi Oyoshi, Naoyuki Hirata

**Affiliations:** Department of Anesthesiology, Kumamoto University Hospital, 1-1-1 Honjo, Chuo-Ku, Kumamoto 860-8556, Japan

## Abstract

**Background:**

Intraoperative anesthetic requirements might be altered due to the modulated metabolic function in living donor liver transplant recipients. Remimazolam may provide appropriate anesthesia in patients with cirrhosis. However, the efficacy and safety of remimazolam in liver transplant recipients have not been reported. We present the successful anesthesia management of a liver transplant recipient using remimazolam. *Case Presentation*. A 54-year-old woman who was diagnosed with Child-Pugh C cirrhosis of unknown etiology was scheduled for living donor liver transplantation. Remimazolam was used for anesthesia management under electroencephalogram monitoring, including bispectral index (BIS) and patient state index (PSI) values. Despite the prolonged surgical time (1,037 min) and massive blood loss (22,500 mL), BIS and PSI values were maintained within acceptable ranges intraoperatively. There was no intraoperative awareness/recall or adverse events associated with remimazolam administered perioperatively.

**Conclusions:**

We safely managed general anesthesia for living donor liver transplantation with remimazolam using electroencephalogram monitoring.

## 1. Background

Living-donor liver transplantation in patients with cirrhosis requires careful anesthesia management. Patients with cirrhosis have circulatory abnormalities due to portal hypertension, which causes systemic hypotension due to splanchnic vasodilation. It also causes sodium and water retention by stimulating the renin-angiotensin-aldosterone system, thereby increasing the patient's plasma volume. This results in unstable hemodynamics with low blood pressure but increased cardiac output [1]. Cirrhosis also reduces the liver's ability to metabolize drugs. Although many drugs are metabolized by cytochrome P450 (CYP), remimazolam is metabolized by human carboxylesterase 1 (HCE1) [2]. In cirrhosis, HCE maintains relatively high functionality relative to CYP, and there have been reports of rapid postoperative arousal following remimazolam use in cirrhotic patients [3], although there have been no reports of remimazolam use during the anhepatic phase in liver transplant recipients.

We report a case in which a patient was safely managed under general anesthesia using remimazolam during a living donor liver transplant.

## 2. Case Presentation

A 54-year-old woman (height 154 cm, weight 53 kg) was scheduled for living donor liver transplantation. The patient was diagnosed with noncompensated cirrhosis of unknown etiology at 53 years of age. Physical examination revealed mild edema of the lower limbs and jaundice of the skin. There were no neurological findings to suggest hepatic encephalopathy, although she presented to the emergency department with lalopathy and bizarre behavior at the age of 54 years and was diagnosed with a left frontal subcortical hemorrhage, for which she received conservative treatment.

Hematological tests showed increased total bilirubin levels (7.1 mg/dL), a low albumin concentration (2.5 g/dL), and a low platelet count (5.7 × 10^4^/*μ*L). Abdominal computed tomography showed ascites and splenomegaly. Based on these tests, her Child-Pugh score was 10 points (grade C). Echocardiography and electrocardiography showed no evidence of reduced cardiac function.

**Figure 1:**
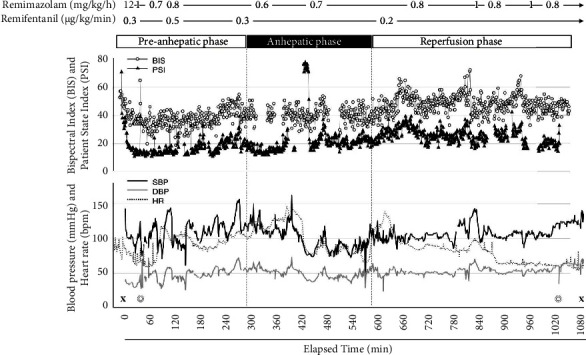
Anesthesia record. SBP: systolic blood pressure; DBP: diastolic blood pressure; HR: heart rate; X, start and end of anesthesia; and ◎, start and end of operation.

Under electroencephalographic monitoring of the bispectral index (BIS) and patient state index (PSI), anesthesia was induced with remimazolam (12 mg/kg/h), remifentanil (0.3 *μ*g/kg/min), and rocuronium (50 mg). The package insert for remimazolam recommends an initial dose of 12 mg/kg/h for induction, followed by 1 mg/kg/h for maintenance of general anesthesia. We adjusted the dose of remimazolam during maintenance on the basis of BIS and PSI. The time to loss of consciousness was about 2 minutes after the start of remimazolam administration. Thus, about 20 mg remimazolam was administered for anesthesia induction. At the time of intubation, her BIS and PSI values were 40–50 and 20–30, respectively. After intubation, the remimazolam infusion rate was reduced to 0.6 mg/kg/h but was increased up to 1.0 mg/kg/h if the BIS value was >60 or PSI value was >50 during surgery (Figure 1).

Intraoperatively, the patient's volume load and noradrenaline and dopamine doses were adjusted to maintain her systolic blood pressure at approximately 100 mmHg. Adequate coagulation function was maintained with transfusions of fresh frozen plasma (FFP), targeting a PT-INR of <2.0, and electrolytes were administered as required. The remimazolam infusion rate was reduced from 0.8 mg/kg/h to 0.6 mg/kg/h in the anhepatic phase. When we replaced a sensor of PSI during the phase, PSI values increased to above 60 for 10 min while the BIS values was 30–50. At that time, bolus doses of remimazolam (0.2 mg/kg) were administered, and the infusion rate was increased to and maintained at 0.7 mg/kg/h. In the reperfusion phase, since both BIS and PSI values increased slightly, the remimazolam infusion rate was increased from 0.8 to 1.0 mg/kg/h. This patient had an abnormal splenic arteriovenous shunt, and a splenic artery injury during surgical manipulation caused massive hemorrhage. Consequently, the surgical duration was 1,037 minutes, and blood loss was approximately 22,500 mL. Remimazolam was stopped following wound closure, and propofol (3 mg/kg/h) and dexmedetomidine (0.4 *μ*g/kg/h) were started. The patient was transferred without extubation to the intensive care unit (ICU). Subsequently, propofol was gradually decreased from 6 hours after surgery. About 12 hours after surgery, after confirming that the patient had recovered from anesthesia and that her breathing and hemodynamics were stable, her trachea was extubated. Although non-invasive positive pressure ventilation (NPPV) was applied for several days postoperatively due to atelectasis, she was transferred from the ICU to the general ward on supplementary nasal oxygen (2 L/min) on postoperative day 5. There was no intraoperative awareness/recall or adverse events associated with remimazolam perioperatively.

## 3. Discussion

Remimazolam is a new benzodiazepine anesthetic available in Japan and Korea for general anesthesia. It has a similar structure to midazolam [4, 5], with the difference being that midazolam is metabolized by CYP and remimazolam by HCE1 [2, 5]. In patients with cirrhosis, the metabolism of midazolam and propofol is reduced because CYP expression in the liver is suppressed [6, 7]. Although Stohr et al. [8] reported that the metabolism of remimazolam might be prolonged in patients with hepatic impairment, Onoda et al. [3] considered that carboxylesterase activity might be preserved in patients with cirrhosis at a level that is sufficient to metabolize remimazolam. In the present case, it is unlikely that metabolism was delayed, as the patient could be extubated without delayed arousal after ICU admission, and resedation was not observed after extubation.

The liver transplantation procedure is divided into preanhepatic, anhepatic, and reperfusion periods. With regard to the preanhepatic phase, we expected a similar hepatic metabolic capacity as in the cases reported by Onoda et al. HCE1, which metabolizes remimazolam, is mainly distributed in the liver, with 60%–70% function remaining in cirrhotic patients [9]. We speculated that the metabolism of remimazolam would likely be reduced and that BIS and PSI values would decrease during the change in the surgical phase from the preanhepatic to the anhepatic phase. Therefore, we reduced the remimazolam infusion rate from 0.8 mg/kg/h to 0.6 mg/kg/h in the anhepatic phase, although BIS and PSI values did not decrease. Despite the remimazolam infusion rate of 0.7 mg/kg/h during most of the anhepatic period in this case, BIS and PSI values were comparable to those in the preanhepatic phase. We therefore speculated that remimazolam continued to be metabolized during the anhepatic phase. In the reperfusion period, since both BIS and PSI values increased slightly, the remimazolam dose rate was increased. This suggests that HCE1 in the graft might have metabolized remimazolam after reperfusion. A previous study reported that genetic regulators can affect HCE1 expression and activity, resulting in the alteration of the metabolism and clinical outcome of HCE 1 substrate drugs [10]. In this liver transplantation, the female recipient received a liver from a male living donor. Thus, genetic factors may induce the differential metabolism of remimazolam among the preanhepatic, anhepatic, and reperfusion phases.

Postoperatively, the patient was sedated with propofol and dexmedetomidine based on our institutional protocol and was extubated without delayed arousal. No resedation after extubation was observed. Hence, it is unlikely that remimazolam affected the postoperative status of the patient.

In living donor liver transplantation, maintaining perioperative hepatic blood flow (HBF) is essential for maintaining normal postoperative liver function [11, 12]. Propofol and sevoflurane have similar effects in the maintenance of HBF, with no difference in the incidence of postoperative graft dysfunction [13, 14]. On the other hand, in a comparative study of remimazolam and propofol, the frequency of intraoperative hypotension was significantly lower in patients receiving remimazolam [15]. Thus, although propofol and sevoflurane are currently used for the management of anesthesia in living donor liver transplant recipients, remimazolam might provide more stable circulatory dynamics in liver transplantation.

## 4. Conclusion

Remimazolam might be an option in the anesthetic management of patients undergoing living donor liver transplantation. Following liver transplantation, the recipient in the present case, who was preoperatively classified as Child-Pugh C, could be extubated without delayed arousal. Future studies focusing on intraoperative blood levels of remimazolam in recipients during living donor liver transplantation will help guide anesthetic management in these patients.

## Data Availability

The data that support the findings of this report are available from the corresponding author, N.H., upon reasonable request.
